# Measuring PERMA+4: validation of the German version of the Positive Functioning at Work Scale

**DOI:** 10.3389/fpsyg.2023.1231299

**Published:** 2023-08-10

**Authors:** Timo Lorenz, Janika Ho, Marla Beyer, Leonie Hagitte

**Affiliations:** Department of Psychology, MSB Medical School Berlin, Berlin, Germany

**Keywords:** validation, PERMA, positive psychology, PERMA+4, wellbeing, work-related wellbeing, positive organizational psychology

## Abstract

This study investigates the association between the PERMA+4 model and psychological safety, while also examining the validation of the Positive Functioning at Work (PFW) scale in a German-speaking population. The study discovered strong association between PERMA+4 and psychological safety, which raises important questions and potential concerns regarding the jangle fallacy. Similar to the PERMA model, PERMA+4 should be considered a framework for attaining psychological safety. The German version of the PFW scale demonstrated satisfactory fit with the model, indicating its factorial validity. To gain insights into promoting workplace wellbeing, it is recommended to conduct longitudinal studies to determine whether psychological safety is a cause or result of PERMA+4. This study enhances our understanding of workplace wellbeing and emphasizes the association between PERMA+4 and psychological safety.

## 1. Introduction

Several researchers have attempted to identify the fundamental components that contribute to high levels of wellbeing (Ryff, 1989a; Diener and Suh, 1997; Ryff and Singer, 2008). One way to conceptualize these components that contribute to wellbeing is through Seligman (2002, 2011) PERMA model.

### 1.1. PERMA

The model consists of five core elements, represented by the acronym PERMA. The five core elements of PERMA are, the focus on experiencing positive emotions such as joy, gratitude, and hope (*Positive Emotions*), being fully engaged and absorbed in activities that provide a sense of flow and purpose (*Engagement*), building positive relationships with others and cultivating a sense of social connection as well as support (*Relationships*), finding purpose and meaning in life through a sense of belonging, personal growth, as well as contribution to something larger than oneself (*Meaning*), and lastly setting and achieving goals, which can provide a sense of accomplishment and competence (*Accomplishment*). Seligman (2011) suggested all of these elements being intrinsically rewarding and in themselves representing goals which lead to human flourishing.

#### 1.1.1. Positive emotions

Positive emotions encompass the occurrence of pleasant feelings such as love, happiness, gratitude, or joy (Donaldson et al., 2020). They are often linked to a general inclination toward satisfaction (Goodman et al., 2018) and are commonly employed as an indicator of wellbeing within the hedonic approach. The balance between positive and negative emotions is frequently utilized to gauge life satisfaction (Bradburn, 1969; Diener, 1984; Diener and Larsen, 1993; Kahneman et al., 1999; Lyubomirsky and Lepper, 1999; Fredrickson and cohn, 2008). The experience of positive emotions is also believed to foster resilience, promote flourishing, and augment happiness and life satisfaction (Bryant, 2003; Cohn et al., 2009; Diener et al., 2009; Silton et al., 2020). Moreover, positive emotions contribute to both physical and mental wellbeing (Alexander et al., 2021).

#### 1.1.2. Engagement

Engagement is a key component of wellbeing, it describes the experience of being fully involved in the activities of one’s life (Donaldson et al., 2020). Flow is a specific type of engagement occurring when individuals are completely immersed in a task. Flow can be a source of great satisfaction and fulfillment (Csikszentmihalyi, 1990; Seligman, 2011; Kun et al., 2017).

The concept of flow is a mental state in which an individual experiences a feeling of “effortless action.” This state is achieved when a person is faced with a high level of challenge that is balanced by possessing the necessary skills to overcome it. As a result, this state leads to deep engagement with a task, which often produces a feeling of concentration that distinguishes it from everyday experiences.

It has been hypothesized that engagement contributes to long-term wellbeing by facilitating the development of positive resources such as talents, honing interests, and practicing skills, which in turn lead to an upwards spiral of wellbeing (Schueller and Seligman, 2010). It has been found in a variety of studies that engagement improves mental wellbeing in work context (e.g., Bakker and Demerouti, 2008; Robertson et al., 2012).

#### 1.1.3. Positive relationships

Positive relationships are a key aspect of human wellbeing. This is because humans are social creatures (Young, 2008), and our wellbeing is linked to our relationships with others. According to research by Goodman et al. (2018) and Donaldson et al. (2020), positive relationships are characterized by feelings of love, appreciation, support, and being valued.

The link between relationships and subjective wellbeing has been extensively researched (see Diener, 1984; Cutrona and Russell, 1987; Diener and Seligman, 2002; Lucas et al., 2003). It is important to include relationships in any model of human wellbeing because of their significant impact on the overall sense of happiness and fulfillment of human beings.

#### 1.1.4. Meaning

Meaning can be defined as a feeling that one’s life has direction and purpose, as well as a connection to something greater. According to Steger (2012), meaning provides a sense of value and worth to one’s life, influencing actions and behaviors. It also helps individuals to establish coherence in their lives by recognizing patterns and establishing predictability (Heintzelman and King, 2014).

Steger (2012) further emphasizes that meaning brings about a sense of importance and purpose to life. It allows individuals to understand how they perceive the world and how they form relationships as a result. Examples of activities that can provide meaning and purpose include belonging to groups or volunteering, as these affiliations contribute to a sense of purpose and value (Kun et al., 2017).

Having meaning in life is linked to indicators of wellbeing such as positive emotions, happiness, life satisfaction (Ryff, 1989b; Steger, 2018), and better psychological adjustment (Ryff, 1989a; O’Conner and Vallerand, 1998; Steger et al., 2006; Steger, 2018). It can also contribute to a positive self-image, higher self-esteem (Ryff, 1989b), and feelings of self-worth (O’Conner and Vallerand, 1998).

In summary, meaning is an important component contributing to wellbeing as it provides a sense of direction, purpose, and coherence in life. It also contributes to the formation of relationships and a greater understanding of the world around us.

#### 1.1.5. Accomplishment

Accomplishment is defined as the mastery of a particular area of interest or the achievement of specific life goals or work objectives (Donaldson et al., 2020). Seligman (2011) suggested that the pursuit of accomplishments may be a goal in itself, as people are often motivated to achieve mastery or proficiency in/of something, even if it does not lead to positive affect, engagement, relationships, or meaning. Accomplishments can enhance wellbeing by increasing internal motivation, self-efficacy, and resilience through the accomplishment of self-set goals.

### 1.2. Critique of the PERMA model

The validity of the PERMA model for measuring wellbeing has been questioned. Critics have argued that the model may not provide a comprehensive measurement strategy for wellbeing, having empirical and theoretical limitations (Goodman et al., 2018; Donaldson et al., 2020). Given the various theories and definitions of wellbeing (see Maslow, 1954, 1971; Rogers, 1961; Shin and Johnson, 1978; Ryff, 1989a; Ryan and Deci, 2001; Dodge et al., 2012), it is unclear whether different models of wellbeing represent distinct types of wellbeing or might be identical, creating a jangle fallacy (Goodman et al., 2018).

Goodman et al. (2018) examined the correlation between the PERMA model and subjective wellbeing (Diener, 1984) and suggested that Seligman’s PERMA model was redundant with Diener’s theory of subjective wellbeing. Seligman (2018) responded to the criticism by clarifying that PERMA should be viewed as an explanatory model rather than a comprehensive theory of wellbeing. The model provides a framework for understanding the building blocks of wellbeing. Seligman also emphasized that while the individual building blocks of the model can be considered independently, they are highly correlated with each other, which forms the basis of the model.

Additionally, Seligman (2018) responded by presenting six criteria aimed at expanding the framework. These criteria were formulated to address the limitations of the original framework, enhancing its effectiveness in promoting wellbeing and guiding interventions. The six criteria are as follows: (a) The new elements must have a direct relation with wellbeing, (b) each element should be pursued as an ultimate goal rather than a means to achieve another goal, (c) the new elements should be capable of leading to developmental interventions, (d) all factors included in the expanded framework should be concise and economical, (e) the framework should remain receptive and adaptable to new advancements in the science of wellbeing, and (f) each new element incorporated into the expanded framework must be independently defined and measured to ensure consistency and clarity.

These criteria ensure that any new elements added to the framework are relevant to wellbeing, can lead to practical interventions, and are easy to understand and to measure. The principle of parsimony ensures that the framework remains simple and accessible, while also explaining wellbeing effectively. The criterion of openness and flexibility acknowledges that the science of wellbeing is still evolving and that new developments may emerge that require the framework to adapt. Finally, the requirement for independent definition and measurement ensures that the framework is clear and consistent in its use.

### 1.3. PERMA+4

Donaldson (2019) discussed the limitations of the PERMA model’s five building blocks in defining wellbeing in all life domains, especially in work contexts. To address this issue, Donaldson (2019) proposed four new building blocks of wellbeing at work. *Physical Health*, *Mindset*, *Work Environment*, and *Economic Security* are the four key factors that can impact individual wellbeing, as well as work performance. Physical health encompasses biological, functional, and psychological health assets and can contribute to an individual’s overall wellbeing and productivity. A growth mindset, characterized by an optimistic and future-oriented perspective, can help individuals view challenges as opportunities to grow, and psychological capital, perseverance, or grit can further strengthen this mindset. The physical work environment includes elements such as access to natural light, fresh air, physical safety, and a positive psychological climate and can also influence an individual’s wellbeing and work performance. Thus, aligning the work environment with the individual’s preferences is crucial. Finally, perceptions of economic security and stability can impact an individual’s satisfaction of their needs and contribute to their overall wellbeing (Donaldson et al., 2020).

The combination of these new building blocks with the PERMA elements formed the PERMA+4 model, also known as the Positive Functioning at Work model (Donaldson et al., 2020; Donaldson and Donaldson, 2021; Cabrera and Donaldson, 2023). The authors emphasized the importance of considering these additional building blocks when discussing wellbeing in the workplace. The new positive functioning framework aims to create multi-component positive psychology interventions that can be used in the workplace to improve employee wellbeing.

The PERMA+4 framework has been empirically investigated, particularly in the development and evaluation of the Positive Functioning at Work (PFW) Scale by Donaldson (2019) and Donaldson and Donaldson (2021). The PFW Scale aims to measure the nine building blocks of wellbeing as outlined above.

### 1.4. Psychological safety

Psychological safety refers to the perception of an environment where there is interpersonal trust and mutual respect, allowing individuals to feel comfortable being themselves and taking interpersonal risks in the workplace (Edmondson, 1999). It’s not related to the perception of one’s work or tasks (Frazier et al., 2017). Without psychological safety, individuals may fear negative consequences on their self-image, status, or career for engaging in learning behaviors such as asking questions, seeking help, experimenting with unknown actions, seeking feedback, or having constructive discussions (Kahn, 1990; Edmondson, 2002). Fear of rejection, incompetence, judgment, or embarrassment may arise during interactions with others (Edmondson and Lei, 2014), furthermore psychosocial consequences such as violence, bullying, and stress in the workplace may occur (Ertel and Stilijanow, 2009). Promoting psychological safety in the workplace is crucial to prevent these negative effects and to promote mutual respect, recognition, and trust.

When psychological safety is present in the workplace, there is a greater chance for innovation, improved performance, and improvement in the team (Edmondson, 2002). Employees also experience higher levels of work satisfaction due to reduced anxiety from the need for self-protection falling away. This, in turn, allows individuals to focus on their work and professional development, and increases creativity (Hackman and Oldham, 1976; Madjar and Ortiz-Walters, 2009). Studies have shown a positive impact of psychological safety on work task performance and increased sharing of information (Baer and Frese, 2003; Bunderson and Boumgarden, 2010; Schaubroeck et al., 2011).

#### 1.4.1. Psychological safety and PERMA+4

Previous research has suggested a possible association between psychological safety and the building blocks of PERMA+4, indicating that they could be related concepts. The importance of positive interpersonal relationships, particularly with leaders, has been highlighted in several studies as having a significant impact on perceptions of psychological safety (Kahn, 1990; Edmondson, 1999; Frazier et al., 2017). Leaders play a crucial role in shaping what is considered safe and acceptable behavior in the workplace, and their actions can determine the likelihood of negative interpersonal consequences for engaging in learning behaviors that promote psychological safety (Edmondson, 1999).

The fostering of psychological safety has also been linked to higher levels of engagement in the workplace (Kahn, 1990; May et al., 2004; Tiwari and Lenka, 2016). Kahn (1990) found that individuals were more likely to personally engage in situations when psychological safety was high. Additionally, individuals who have a learning orientation, which is similar to the concept of growth mindset included in the PERMA+4 model, exhibit a positive association with feelings of psychological safety (Chiu et al., 2011).

While the element of accomplishment has not been explicitly linked to psychological safety in previous literature, pursuing shared goals has been found to provide direction and motivation to teams and reduce the need for self-protection (Edmondson, 2002). When goals are shared or interdependent, it enables problem-solving efficiency in groups and promotes more communication of ideas and information, as well as openness to the views of others in the team (Tjosvold, 1990), which could result in higher levels of psychological safety.

### 1.5. Aim of this study

The PFW Scale is a self-report measure consisting of 29 items that assess wellbeing. Previous results in US samples (Donaldson et al., 2020) show that both a nine first-order factorial model and a hierarchical second-order model, which is composed of nine first-order factors, effectively measure the nine building blocks and exhibit various forms of validity. The PFW Scale exhibits convergent, discriminant, criterion, predictive, and incremental validity when compared with other forms of wellbeing, such as satisfaction with life (Diener et al., 1985) and PsyCap (Luthans et al., 2007), and performance measures such as positive work role performance (Griffin et al., 2007).

The aim of this study is to validate and translate the PFW (Donaldson and Donaldson, 2020) for use with German-speaking individuals. The PFW was developed as a tool for assessing Positive Functioning at Work. By validating the German version of the PFW, this study aims to provide a reliable measure for assessing positive functioning in the workplace for German-speaking individuals.

To achieve this objective, the psychometric properties of the German version of the PFW will be assessed and validated. This study aligns with the call to action for better measurement validation in positive psychology (van Zyl et al., 2023) and psychology as a whole (Flake and Fried, 2020).

## 2. Methods

In this section, we present the results for all the outcome variables that were assessed. No additional dependent variables were included or excluded during the course of this study.

### 2.1. Participant recruitment and survey

The sample consisted of 379 participants. In the sample 138 (36.41%) participants identified as male, 240 (63.32%) participants identified as female, and one individual (0.26%) identified as diverse. The mean age of the participants was 34.9 years old (SD = 12.7). The participants were asked to choose their category of profession from the classification of occupations (Bundesagentur für Arbeit, 2011). The majority of participants worked in the field of “health, social care, teaching and education” (37.20%). Further, the participants were asked about their highest level of completed education. Most of the participants were highly educated, meaning that they had received a secondary education of some sort (Bachelor or equivalent, masters, etc.).

The participants for this study were recruited through various methods to ensure a diverse sample. Recruitment efforts were primarily focused on utilizing social networks and online platforms to reach potential participants. Advertisements and invitations were shared through social media platforms, such as LinkedIn. In addition to online recruitment, offline strategies were employed to broaden the reach of the study. QR codes were distributed in public places, including parks and train stations, allowing individuals passing by to scan the code and access the study information.

The study was conducted in German and participation was voluntary, hence no incentives were supplied. Due to forced choice in the standardized questionnaires, there was no missing data.

### 2.2. Instruments

#### 2.2.1. Positive Functioning at Work

Positive Functioning at Work, as conceptualized within the PERMA+4 model, was assessed using the Positive Functioning at Work Scale (Donaldson et al., 2022). This instrument consists of 30 items, rated on a 7-point scale ranging from 1 = “strongly disagree” to 7 = “strongly agree.” In the present study, McDonald’s omega ω_t_ was found to be 0.98, indicating high internal consistency, and Cronbach’s α was 0.94, suggesting good reliability.

To ensure an accurate translation of the Positive Functioning at Work (PFW) Scale into German, a rigorous translation process was followed. Initially, the items were translated from English to German, with careful consideration given to possible variations in translation. Subsequently, a bilingual German-English speaker performed a back-translation of the German version. Finally, the initial and final translations were reviewed by a native English speaker who possesses a German high school diploma, ensuring the accuracy and fidelity of the translations.

Additionally, as part of the translation process, an additional item was created: “My income allows me to provide financially for my future.” This decision was made because the item “I could lose several months of pay due to serious illness, and still have my economic security.” may have less relevance in German-speaking countries, considering the presence of statutory health insurance. Table 1 provides a summary of both the original items and their corresponding German translations.

**Table 1:** Item wordings and abbreviations of the Positive Functioning at Work Scale.

Dimension	English (from Donaldson et al., 2022)	German	Item abbreviation
Positive emotions	I feel joy in a typical workday.	An einem typischen Arbeitstag empfinde ich Freude.	p1
	Overall, I feel enthusiastic about my work.	Alles in allem begeistert mich meine Arbeit.	p2
	I love my job.	Ich liebe meinen Job.	p3
Engagement	I typically become absorbed while I am working on something that challenges my abilities.	In der Regel bin ich sehr vertieft, wenn ich an etwas arbeite, das meine Fähigkeiten herausfordert.	e1
	I lose track of time while doing something I enjoy at work.	Ich verliere das Zeitgefühl, wenn ich bei der Arbeit etwas tue, was mir Spaß macht.	e2
	When I am working on something I enjoy, I forget everything else around me.	Wenn ich an etwas arbeite, das mir Spaß macht, vergesse ich alles andere um mich herum.	e3
Relationships	I can receive support from coworkers if I need it.	Ich kann von meinen Kolleginnen und Kollegen Unterstützung erhalten, wenn ich sie brauche.	r1
	I feel appreciated by my coworkers.	Ich fühle mich von meinen Kolleginnen und Kollegen wertgeschätzt.	r2
	I trust my colleagues.	Ich vertraue meinen Kolleginnen und Kollegen.	r3
	My colleagues bring out my best self.	Meine Kolleginnen und Kollegen holen das Beste aus mir heraus.	r4
Meaning	My work is meaningful.	Meine Arbeit ist bedeutsam.	m1
	I understand what makes my job meaningful.	Ich weiß, was meine Arbeit bedeutsam macht.	m2
	The work I do serves a greater purpose.	Meine Arbeit dient einem höheren Zweck.	m3
Accomplishment	I set goals that help me achieve my career aspirations.	Ich setze mir Ziele, die mir dabei helfen, meine Karrierewünsche zu erreichen.	a1
	I typically accomplish what I set out to do in my job.	In der Regel erreiche ich, was ich mir in meinem Job vorgenommen habe.	a2
	I am generally satisfied with my performance at work.	Im Allgemeinen bin ich mit meiner Leistung bei der Arbeit zufrieden.	a3
Physical health	I typically feel physically healthy.	Normalerweise fühle ich mich körperlich gesund.	ph1
	I am rarely sick.	Ich bin selten krank.	ph2
	I can typically overcome sources of physical distress (e.g., insomnia, injuries, and vision issues).	Normalerweise kann ich körperliche Beschwerden bewältigen (z.B. Schlaflosigkeit oder Verletzungen).	ph3
	I feel in control of my physical health.	Ich habe das Gefühl, meine körperliche Gesundheit unter Kontrolle zu haben.	h4
Mindset	I believe I can improve my job skills through hard work.	Ich glaube, dass ich meine beruflichen Kompetenzen durch harte Arbeit verbessern kann.	mi1
	I believe my job will allow me to develop in the future.	Ich glaube, dass mein Job mir die Möglichkeit gibt, mich in Zukunft weiterzuentwickeln.	mi2
	I have a bright future at my current work organization.	Ich habe eine vielversprechende Zukunft in meiner derzeitigen organization.	mi3
Environment	My physical work environment (e.g., office space) allows me to focus on my work.	Mein physisches Arbeitsumfeld (z. B. Büroräume) ermöglicht es mir, mich auf meine Arbeit zu konzentrieren.	en1
	There is plenty of natural light in my workplace.	An meinem Arbeitsplatz gibt es viel natürliches Licht.	en2
	I can conveniently access nature in my work environment (e.g., parks, oceans, and mountains).	Ich habe in meinem Arbeitsumfeld leicht Zugang zur Natur (z. B. zu Parks, dem Meer und Bergen).	en3
Economic security	I am comfortable with my current income.	Ich bin zufrieden mit meinem derzeitigen Einkommen.	ec1
	I could lose several months of pay due to serious illness, and still have my economic security. *Alternative Item:* My income allows me to provide financially for my future.	Ich könnte mehrere Monate Einkommen durch eine schwere Erkrankung verlieren und hätte trotzdem finanzielle Sicherheit. *Alternatives Item:* Mein Einkommen erlaubt es mir, finanziell für meine Zukunft vorzusorgen.	ec2
	In the event of a financial emergency, I have adequate savings.	Für finanzielle Notfälle hätte ich ausreichend Ersparnisse.	ec3

#### 2.2.2. PERMA

The assessment of PERMA utilized the PERMA-Profiler (Butler and Kern, 2016; Wammerl et al., 2019), employing an 11-point rating scale ranging from 0 = “never” to 10 = “always” or 0 = “not at all” to 10 = “completely.” Alongside the 15 PERMA items (e.g., “How often do you become absorbed in what you are doing?”), the instrument also included eight filler items. A higher composite score indicates a greater level of wellbeing. In the present study, McDonald’s omega (ω_t_) was calculated as 0.98, and Cronbach’s α was 0.95.

#### 2.2.3. Psychological safety

The assessment of workplace psychological safety was conducted using the PsySafety-Check (PS-C) (Fischer and Hüttermann, 2020). The scale consisted of 7 items (e.g., “When working together in this team, my special abilities and talents are valued and used.”), employing a 7-point rating scale ranging from 1 = “strongly disagree” to 7 = “strongly agree.” In this study, the calculated McDonald’s omega (ω_t_) for the PS-C was 0.87, and Cronbach’s α was 0.86.

### 2.3. Analysis

Confirmatory factor analyses (CFAs) were conducted using maximum likelihood robust estimation (MLR) to test the factorial validity of the PFW scale, considering violations of normality distribution (Beauducel and Herzberg, 2006). The objective was to replace one item from the original scale and compare the two versions in terms of model fit. The measurement model consisted of 29 items, with nine first-order factors (positive emotions, engagement, relationships, meaning, accomplishment, physical health, mindset, environment & economic safety) and one second-order factor. To assess the fit of the measurement model, the criteria for an acceptable fit proposed by Gäde et al. (2020) were applied, which included a standardized root-mean-square residual (SRMR) ≤ 0.10, root-mean-square error of approximation (RMSEA) ≤ 0.08, lower bound of the 90% confidence interval of RMSEA ≤0.08, comparative fit index (CFI) ≥ 0.90, or Tucker Lewis index (TLI) ≥ 0.90. CFAs and reliability measures were performed using the lavaan package (version 0.6–12; Rosseel, 2012) in R statistical software (version 4.2.2; R Core Team, 2014), with other packages also utilized. Descriptive data included two estimates of internal consistency: omega total (ω_t_; McDonald, 1999) and Cronbach’s coefficient α (Cronbach, 1951). Omega total estimates the overall reliability of a test that does not meet the assumption of τ-equivalence (Revelle and Condon, 2019), while Cronbach’s coefficient α is reported for comparability purposes. To examine potential changes in the construct and account for convergent validity with the PERMA-Profiler, correlations among the latent factors of the different measures were investigated. Furthermore, we examined the latent correlations of PERMA+4, PERMA and psychological safety.

## 3. Results

### 3.1. Measurement model fit indices

Table 2 presents the model fit indices for both the new item-added version of the Positive Functioning at Work (PFW) scale and the original PFW model. Noteworthy are the following indices observed in the new model: Comparative Fit Index (CFI) = 0.93, Tucker-Lewis Index (TLI) = 0.92, Root Mean Square Error of Approximation (RMSEA) = 0.06, 90% Confidence Interval (CI) for RMSEA = [0.05, 0.06], and Standardized Root Mean Square Residual (SRMR) = 0.08. These values closely align with the corresponding indices for the original model, which are CFI = 0.93, TLI = 0.92, RMSEA = 0.06, 90% CI for RMSEA = [0.05, 0.06], and SRMR = 0.08. Descriptive statistics for the nine facets of the PFW scale are provided in Table 3.

**Table 2:** Measurement models using MLM estimator.

	N factors	χ^2^	df	*p*	CFI	TLI	SRMR	RMSEA	RMSEA 90%-KI
PERMA+4 new item	9 + g	731.69	368	<0.001	0.93	0.92	0.077	0.055	0.05–0.06
PERMA+4 original	9 + g	729.60	368	<0.001	0.93	0.92	0.075	0.055	0.05–0.06
PERMA profiler	5 + g	245.07	85	<0.001	0.92	0.90	0.055	0.079	0.07–0.09
Psych. safety	1	39.73	14	<0.001	0.97	0.96	0.034	0.076	0.05–0.10

**Table 3:** Descriptive data of the PFW facets.

	Mean	SD	Skew	Kurtosis
Positive emotions	4.8	1.5	−0.56	−0.48
Engagement	5.2	1.3	−0.60	−0.18
Relationships	5.0	1.3	−0.81	0.29
Meaning	5.1	1.6	−0.70	−0.33
Accomplishment	5.0	1.1	−0.55	0.51
Physical health	5.3	1.3	−0.78	0.14
Mindset	4.7	1.5	−0.42	−0.57
Environment	4.6	1.5	−0.42	−0.48
Economic Security_new	4.2	1.7	−0.16	−1.1
Economic Security_old	4.1	1.8	−0.05	−1.1

Additionally, the model fit of the PERMA-Profiler and the Psychological Safety Climate (PS-C) measures was examined. The PERMA-Profiler yielded the following fit indices: CFI = 0.92, TLI = 0.90, RMSEA = 0.079, 90% CI for RMSEA = [0.07, 0.09], and SRMR = 0.055. Similarly, the PS-C demonstrated favorable fit indices with CFI = 0.97, TLI = 0.96, RMSEA = 0.076, 90% CI for RMSEA = [0.05, 0.10], and SRMR = 0.034.

The covariances of the nine latent first-order factors of the PERMA+4 are summarized in Table 4. It is important to note that these covariances are estimated without considering the second-order factor. This information provides insights into the relations among the individual factors.

**Table 4:** Covariances of PERMA +4 [old version] factors without the hierarchical g-factor.

	Engagement	Relationships	Meaning	Accomplishment	Physical health	Mindset	Environment	Economic security
Positive emotions	0.27 [0.27]	0.56 [0.56]	0.67 [0.67]	0.56 [0.56]	0.33 [0.32]	0.67 [0.67]	0.49 [0.49]	0.25 [0.24]
Engagement		0.22 [0.22]	0.16 [0.16]	0.14 [0.14]	0.17 [0.17]	0.26 [0.26]	0.18 [0.18]	0.04 [0.04]
Relationships			0.38 [0.38]	0.45 [0.45]	0.41 [0.41]	0.50 [0.50]	0.47 [0.47]	0.32 [0.24]
Meaning				0.42 [0.42]	0.23 [0.23]	0.52 [0.52]	0.42 [0.42]	0.17 [0.13]
Accomplishment					0.46 [0.46]	0.53 [0.52]	0.44 [0.44]	0.27 [0.26]
Physical health						0.26 [0.26]	0.37 [0.37]	0.34 [0.35]
Mindset							0.58 [0.58]	0.34 [0.24]
Environment								0.29 [0.30]

### 3.2. Factor loadings

For the PFW with the original questions, the following factor loadings are to be highlighted: the loadings of the second-order factors on Perma +4 are Positive emotions = 0.86, Engagement = 0.30, Relationships = 0.66, Meaning = 0.68, Accomplishment = 0.67, Physical health = 0.46, Mindset = 0.77, Environment = 0.65, and Economic security = 0.32. Of the item loadings onto the first-order factors, regarding the original version, only the loading of the items a1 = 0.42 on the Accomplishment factor and of the item p2 = 0.94 on the Positive emotions factor are to be emphasized, being the highest and the lowest of all first-level-loadings.

**Figure 1:**
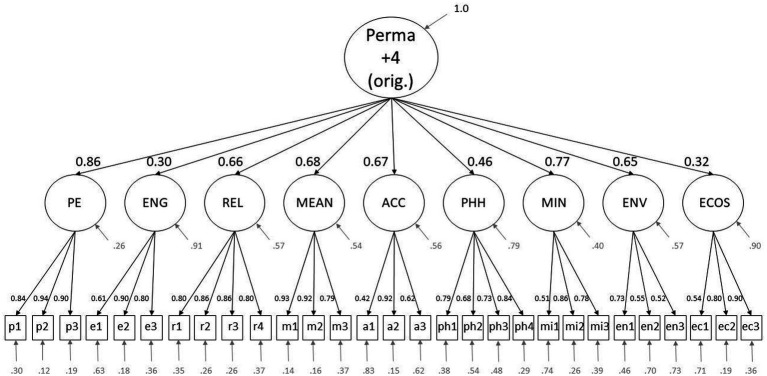
This figure displays the measurement model of the original PFW, showing standardized factor loadings and variances. The abbreviations p1-3, e1-3, r1-4, m1-3, a1-3, ph1-4, mi1-3, en1-3, and ec1-3 represent the items of the scale. The Letters of the abbreviations indicate which is the respective latent factor, PE, Positive Emotions; ENG, Engagement; REL, Relationships; MEAN, Meaning; ACC, Accomplishment; PHH, Physical Health; MIN, Mindset; ENV, Environment; ECOS, Economic Security.

**Figure 2:**
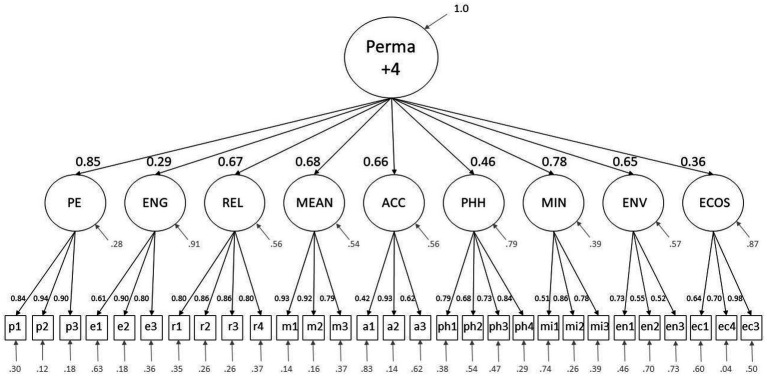
This figure displays the measurement model of the new PFW, showing standardized factor loadings and variances. The abbreviations p1-3, e1-3, r1-4, m1-3, a1-3, ph1-4, mi1-3, en1-3, and ec1-3 represent the items of the scale. The Letters of the abbreviations indicate which is the respective latent factor, PE, Positive Emotions; ENG, Engagement; REL, Relationships; MEAN, Meaning; ACC, Accomplishment; PHH, Physical Health; MIN, Mindset; ENV, Environment; ECOS, Economic Security.

The changes in loadings to highlight in the sample with the new item are as follows: Economic security = 0.36. Furthermore, the item loadings onto the respective first-order factors of the items ec1 = 0.64, ec2 = 0.70, ec3 = 0.98 stand out. The measurement model including standardized loadings of the older version is displayed in Figure 1, and the respective model for the newer version, with the exchanged item, is shown in Figure 2.

Standardized loadings of the second-order factor PERMA+4, ranging from 0.30 to 0.86 (including the new item with a range of 0.30–0.85), may be considered small for some factors. However, it is important to note that despite their size, all loadings were statistically significant with *p* < 0.001. This indicates a meaningful relation between the factors and the second-order factor PERMA+4, emphasizing the importance of these variables in assessing wellbeing.

### 3.3. Standardized latent factor correlations

The correlation coefficients of the latent second-order factors yielded the following results: the correlation between the PERMA-Profiler and the PERMA+4, including the new item, was *r* = 0.71 (*r* = 0.72 with the original items). The study also examined the correlations of both scales with the latent construct of psychological safety. The correlation between the PERMA+4 and psychological safety was *r* = 0.81 (*r* = 0.80 with the original items), while the correlation between the PERMA-Profiler and psychological safety was *r* = 0.37.

## 4. Discussion

In this study, we aimed to translate and validate the PFW (Donaldson and Donaldson, 2021) Scale in a German sample consisting of 379 participants. To determine the adequacy of the PFW-G model, we applied the Gäde et al. (2020) criteria to the CFA results. Despite the significant chi-square test, which may reflect the large sample size, the CFA results suggest that the PFW-G model is an acceptable fit for the data.

We also evaluated the impact of replacing one of the PFW items: “I could lose several months of pay due to serious illness, and still have my economic security.” Our analysis showed that this replacement had little effect on the overall model, indicating that the item should be retained in its original form. Although we considered an alternative item, “My income allows me to provide financially for my future,” we concluded that it would make it harder to compare our findings with the original PFW values. Therefore, we suggest keeping the original item in the PFW.

### 4.1. PERMA+4 and psychological safety

The observed high correlation between PERMA+4 and psychological safety raises important questions for research in the field of positive psychology. This relation echoes the previous debate on the possible jangle fallacy between PERMA and subjective wellbeing (Goodman et al., 2018), which was addressed by Seligman (2018) who argued that PERMA is not a framework for defining wellbeing, but rather a set of elements that are necessary for achieving it.

In their study, Donaldson et al. (2022) propose that psychological safety may be an antecedent for PERMA+4, but we would argue that it is more likely to be an outcome. Consistent with Seligman (2018) view, we see the PERMA+4 model as a framework for promoting wellbeing at work and psychological safety. As such, PERMA+4 can be viewed as building blocks for achieving psychological safety.

However, while the authors acknowledge the strong correlation between PERMA+4 and psychological safety, they rightly point out the need for more theory and description to strengthen the discussion about cause and effect. The question of whether psychological safety is an antecedent or an outcome of PERMA+4 remains an open one that requires further investigation.

To gain a deeper understanding of the relation between psychological safety and PERMA+4, longitudinal studies are necessary. These studies could evaluate the impact of promoting PERMA+4 or psychological safety on the other over time. By examining how changes in one variable affect the other, researchers can shed light on the complex dynamics and potential causal pathways between psychological safety and PERMA+4.

Such findings would not only contribute to the theoretical understanding of the relation but also provide valuable insights for practitioners and researchers alike in promoting wellbeing in the workplace. Understanding whether interventions targeting psychological safety lead to improvements in PERMA+4 or vice versa would have practical implications for organizations seeking to create positive work environments and enhance employee wellbeing.

Furthermore, future research should aim to deepen our understanding of the conceptual differences and potential overlaps between PERMA+4 and psychological safety. One avenue for investigation is to conduct comparative analyses of the underlying theoretical frameworks and operational definitions of these constructs. By critically examining the core components and measurement approaches of each concept, future research could identify similarities, divergences, and areas of potential convergence. Additionally, qualitative studies could provide valuable insights into individuals’ perceptions and experiences of both PERMA+4 and psychological safety, allowing for a richer exploration of their distinct characteristics and potential shared elements. Additionally, empirical studies could explore the boundary conditions under which these constructs exert their influence, investigating whether there are certain contexts or organizational factors that moderate their effects.

### 4.2. Outlook

This study represents the first validation of the PFW instrument in a German-speaking population. We encourage further research to validate the instrument in different languages and cultural contexts. Additional research could incorporate data from other regions such as Europe, Africa, Asia, and South America. Furthermore, standardization of the PFW instrument is necessary to provide contemporary and country-specific norms for meaningful score interpretation. This would allow individuals to evaluate their overall PERMA+4 and identify areas for targeted positive workplace intervention. Building multi-component positive psychological interventions around each model component could help identify the most effective interventions in practice (Donaldson and Chen, 2021).

Moreover, integrating artificial intelligence into the development and implementation of these interventions could offer a promising approach to enhance employee wellbeing and work performance. AI-powered tools could tailor interventions to individual employees based on their unique strengths and needs related to each model component and analyze data to optimize intervention design and delivery. Evaluating this approach at different levels, such as the employee, leadership, group or team, and organizational levels, could identify the most effective interventions and inform future research on improving employee wellbeing and work performance.

Finally, exploring the effects of PERMA+4 on specific outcomes related to the performance of individuals, groups, and organizations could reinforce its efficacy in the literature and provide a strong business rationale for integrating it into organizational practices.

### 4.3. Limitations

During the discussion of our study results, we acknowledge several limitations that could impact the generalizability of our findings. Firstly, the recruitment of participants through personal and professional social networks resulted in a non-probability sample, which raises concerns about generalizability. However, this approach enhanced response rates and provided access to individuals from diverse backgrounds. While online recruitment may have limited our ability to achieve a representative sample, existing research suggests that this method does not significantly affect results (Gosling et al., 2004).

Another limitation is our reliance solely on self-report data for both studies, which introduces the potential for inherent bias. Nevertheless, some argue that self-report data can be reliable (Chan, 2009), and we believe that individuals are the most knowledgeable source when it comes to their own lived experiences. Additionally, the use of self-report measures for all variables simultaneously raises the risk of common method bias (Podsakoff et al., 2003). However, some scholars challenge the assumption that this bias simplifies the issue and distorts the true correlation (Spector, 2006; Bozionelos and Simmering, 2022).

One additional limitation of our study lies in the potential problems associated with the hierarchical g-factor model, which serves as the theoretical foundation for the PERMA and PERMA+4 models. The hierarchical g-factor model has been subject to scrutiny and alternative perspectives have been proposed (e.g., Eid et al., 2017, 2018). It is important to acknowledge that this model may have its own limitations and assumptions that could influence the interpretation of our findings.

One significant limitation is that assessing the hypothesis that the PERMA+4 factors can serve as foundational elements for psychological safety necessitates longitudinal data. Long-term interventions are required to modify these factors in a targeted manner and ascertain which factors potentially influence psychological safety.

## Data availability statement

The raw data supporting the conclusions of this article will be made available by the authors, without undue reservation.

## Ethics statement

Ethical review and approval was not required for the study on human participants in accordance with the local legislation and institutional requirements. The patients/participants provided their written informed consent to participate in this study.

## Author contributions

TL contributed to conception and design of the study. TL, JH, MB, and LH wrote the first draft of the manuscript. TL and LH organized the database and performed the statistical analysis. All authors contributed to manuscript revision, read, and approved the submitted version.

## Conflict of interest

The authors declare that the research was conducted in the absence of any commercial or financial relationships that could be construed as a potential conflict of interest.

## Publisher’s note

All claims expressed in this article are solely those of the authors and do not necessarily represent those of their affiliated organizations, or those of the publisher, the editors and the reviewers. Any product that may be evaluated in this article, or claim that may be made by its manufacturer, is not guaranteed or endorsed by the publisher.
